# Frailty in Stroke Care in Germany Between 2016 and 2022—A Retrospective, Hospital-Based Nationwide Cohort Study

**DOI:** 10.3390/neurosci6030088

**Published:** 2025-09-08

**Authors:** Julius Dengler, Bassam Abdullah, Juraj Kukolja, Ralf Kuhlen, Sven Hohenstein, Nora F. Dengler, Andreas Bollmann, Frederick Palm

**Affiliations:** 1 Faculty of Health Sciences Brandenburg, Brandenburg Medical School Theodor Fontane, Campus Bad Saarow, 15526 Bad Saarow, Germany; 2 Department of Neurosurgery, Helios Hospital Bad Saarow, 15526 Bad Saarow, Germany; 3 Department of Neurology, Helios University Hospital Wuppertal, 42283 Wuppertal, Germany; 4 Faculty of Health, Witten/Herdecke University, 58455 Witten, Germany; 5 Helios Health GmbH, 10117 Berlin, Germany; 6 Real World Evidence and Health Technology Assessment, Helios Health Institute, 13125 Berlin, Germany; sven.hohenstein@helios-gesundheit.de (S.H.);; 7 Department of Electrophysiology, Heart Center Leipzig, 04289 Leipzig, Germany; 8 Department of Neurology, Helios Hospital Schleswig, 24837 Schleswig, Germany

**Keywords:** frailty, Hospital Frailty Risk Score, acute ischemic stroke

## Abstract

This study examines changes in frailty among patients hospitalized for acute ischemic stroke (AIS) in a nationwide hospital cohort in Germany. Data from AIS patients were compared between the period before the corona virus disease 2019 (COVID-19)-pandemic (1 January 2016 to 31 December 2019) vs the pandemic phase (1 January 2020 to 31 December 2022). Frailty was categorized using the Hospital Frailty Risk Score (HFRS). Inferential statistics were conducted using generalized linear mixed models. Among the 101,124 included AIS patients, the median HFRS decreased from 9.3 (interquartile range [IQR]: 5.2–15.5) in pre-pandemic years to 8.4 (IQR: 4.4–14.2) during the pandemic (*p* < 0.01). Among high frailty AIS patients, length of stay rose from 15.7 (±14.9) to 16.0 (±15.0) days, differing significantly from the decrease observed among low frailty patients from 5.9 (±3.7) to 5.0 (±3.5; *p* < 0.01) days. Compared to pre-pandemic levels, among low frailty patients, there was a significant increase in rates of thrombolysis (odds ratio [OR] 1.14 [95% CI 1.02–1.28; *p* = 0.020]) and thrombectomy (OR 1.35 [1.32–1.48; *p* = 0.047]). In this nationwide study in Germany, there was a longitudinal decrease in frailty among patients hospitalized for AIS which was accompanied by increased rates of thrombolysis and thrombectomy.

## 1. Introduction

Frailty is an age-dependent syndrome defined by high vulnerability to low-power stressors and multimorbidity which is prevalent in at least one fourth of all acute ischemic stroke (AIS) patients [1,2,3,4,5].

Current broad scale clinical evidence on associations between frailty and AIS care predominantly utilizes the Hospital Frailty Risk Score (HFRS), an instrument introduced specifically to assess frailty levels among large patient cohorts [3,4,6,7,8]. The HFRS evaluates a predefined set of administrative data, thereby making individual manual patient examination obsolete for frailty assessment [9]. This main advantage of the HFRS makes it a valid tool in frailty assessment, in addition to showing fair correlation with the manually established Clinical Frailty Scale (CFS). Among patients with stroke, the HFRS is associated with outcomes [10], including mRS and length of stay, and numerous in-hospital processes [3,4,11]. Furthermore, the HFRS is associated with the risk of stroke [12] as well as the effectiveness of anticoagulation treatment in preventing stroke among frail individuals [13].

Over recent years, increasing frailty levels have been posing growing demands on stroke care in the context of ever-aging societies [14]. Yet, systematic longitudinal data on frailty levels among patients hospitalized for AIS is lacking. This is noteworthy, given that the assessment of frailty among hospitalized AIS patients may facilitate prognostication [4] and the recent COVID-19 pandemic and associated lockdown measures not only produced substantial interruptions in stroke care [15] but also led to increased frailty among community-dwelling elderly [16,17]. Therefore, a detailed analysis of continuous large-scale trends in the relationship between frailty and stroke over recent years is of interest. The main objective of this study was to delineate longitudinal changes in frailty, quantified by the HFRS, among patients hospitalized for AIS between years 2016 and 2022 in a nationwide network of 78 hospitals in Germany.

## 2. Materials and Methods

### 2.1. Study Design and Data Flow

We used administrative data from 78 Helios hospitals in Germany involved in AIS management within the Helios network, which manages 7% of all in-hospital cases in the country at centers in rural and urban regions in 13 of the 16 federal states [18]. These 78 hospitals are all of the Helios hospitals in Germany, in which AIS patients were hospitalized during the study period between 1 January 2016 and 31 December 2022 with the primary diagnosis of AIS. The following periods were examined separately: pre-pandemic (1 January 2016–31 December 2019) and pandemic (1 January 2020–31 December 2022). AIS was identified according to the International Classification of Diseases, 10th Revision (ICD-10), using the main codes I63.0–I63.9. Stroke management was stratified based on Operations and Procedures codes [OPS (German adaptation of the International Classification of the Procedures in Medicine of the World Health Organization, version 2017)]. The following OPS codes were used as covariates: thrombolysis: 8-020.8, 8-020.d, 8-836.70, 8-836.71; thrombectomy: 8-836.80, 8-836.81; decompressive craniectomy: 5-012.0, 5-010.00-.03, 5.010.10-13; mechanical ventilation: 8-70x, 8-71x or duration of ventilation > 0; and stroke unit treatment among patients without mechanical ventilation: 8-981x. We examined length of stay (LOS) according to full days spent in hospital, and the HFRS based on ICD-10-Codes, as previously described [9]. Also as previously described [9], the following frailty groups were examined: low frailty (HFRS below 5 points), intermediate frailty (HFRS 5 to 15 points), and high frailty (HFRS above 15 points).

Stroke comorbidities, including congestive heart failure, cardiac arrhythmias, peripheral vascular disorder, hypertension, diabetes mellitus, and obesity were tracked based on encoded secondary diagnoses and used to calculate the Elixhauser comorbidity Index (ECI), as previously described [19]. To minimize potential bias due to coding errors, all ICD-10 and OPS codes undergo rigorous screening by in-hospital auditors as a routine measure prior to entry into the hospital system’s database. All data were pseudonymized, and data management was conducted according to national data protection standards. Informed consent was waived due to the retrospective nature of this study. The study was approved by the ethics committee of the University of Leipzig on 7 February 2022 (490/20-ek).

### 2.2. Data Analysis

Administrative data were gathered using QlikView software (QlikTech, Radnor, PA, USA, version 12.2). Inferential statistics were conducted using generalized linear mixed models (GLMM) with hospitals as random factor [20]. Given that we compared two time periods (prepandemic vs. pandemic), time was examined as a binary variable. Effect estimation was done using the R environment (version 4.0.2, 64-bit build) and its lme4 package (version 1.1-21) [21,22]. Varying intercepts were defined for the random factor. A two-tailed 5% error criterion for significance was in place for all tests. Weekly admission trends were described using incidence rate and linear regression models. Patient characteristics were assessed by χ2-tests for binary variables and analysis of variance for numeric variables. Rates of treatments and outcomes were examined by GLMMs with logit link function. To compare different frailty groups, high frailty was defined as reference. Daily case numbers and frailty scores were examined using negative binomial model. To produce integer values for this analysis, scores were multiplied with ten. Ratios with 95% confidence intervals (CI) were established by exponentiation of the regression coefficients. Frailty groups were used as treatment contrasts (low vs. high, intermediate vs. high) and periods were specified as 0.5 (pandemic) vs. −0.5 (pre-pandemic). Length of stay (LOS) was examined using an LMM based on a log-transformed dependent variable.

## 3. Results

### 3.1. Changes in Admissions in Relation to Frailty

A total of 101,124 patients with AIS were hospitalized between 2016 and 2022, all of which were included in the study.

**Figure 1:**
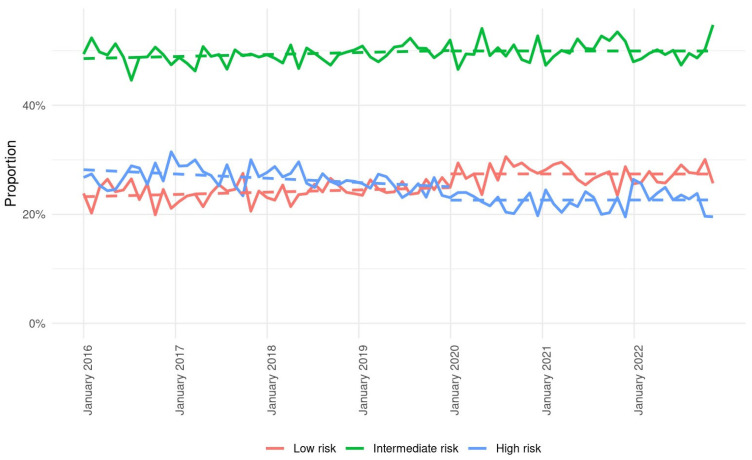
Weekly admissions for stroke during pre-pandemic and pandemic periods according to frailty levels low (red line), intermediate (green line), and high (blue line). Dashed lines represent trends in weekly admission based on incidence rate and linear regression models.

A detailed depiction of frailty trends is presented in Figure 1. The total numbers and percentages of patients in prepandemic/pandemic periods for low frailty were 14,280 (24.0%)/11,474 (27.5%), for intermediate frailty 29,260 (49.3%)/20,888 (50.0%), and for high frailty 15,837 (26.7%)/9385 (22.5%). Most notably, between years 2016 and 2022, among admitted AIS patients, the proportions of low and high frailty show opposite trends, with a consistent decrease in high versus an increase in low frailty.

As displayed in Table 1, average daily admissions of patients with AIS decreased from 40.6 ± 9.1 during the pre-pandemic period to 38.4 ± 8.4 during the pandemic period (*p* < 0.01).

At the same time, the median HFRS decreased from 9.3 (IQR: 5.2–15.5) in pre-pandemic years to 8.4 (IQR: 4.4–14.2) during the pandemic period (*p* < 0.01). Daily admissions were significantly associated with frailty levels.

### 3.2. Changes in Baseline Characteristics in Relation to Frailty

Table 1 shows no significant age differences during the pandemic versus pre-pandemic years (*p* = 0.25). The proportion of male patients increased significantly during the pandemic (51.8% vs. 52.9%; *p* < 0.01). The mean ECI decreased from 10.5 (±10.3) in the pre-pandemic period to 9.8 (±9.9) during the pandemic (*p* < 0.01). Trends in ECI were significantly associated with frailty, given that high frailty risk patients showed increasing ECI (from 18.4 to 18.6) versus decreasing ECI among intermediate frailty patients (9.8 to 9.6; *p* < 0.01). More detailed analyses stratified for age and sex can be viewed in the Supplementary Table S1.

The average LOS was significantly shorter in the pandemic period (9.1 days) compared with pre-pandemic levels (10.1 days, *p* < 0.01, Table 2). Trends in LOS were associated with frailty, given that, with high frailty as reference, in which there was an increase in LOS, changes among intermediate and low frailty patients differed significantly in that they both showed decreasing LOS (each *p* < 0.01).

### 3.3. Changes in Rates of Treatment and In-Hospital Mortality in Relation to Frailty

Among all examined treatment types and in-hospital processes, significant frailty-associated alterations, compared to pre-pandemic levels, were observed only in rates of thrombolysis and thrombectomy (Table 3). For both of those interventions, with high frailty patients as reference, rates increased more substantially among those with low frailty levels, with odds ratios of 1.14 (95% CI, 1.02–1.28; *p* = 0.020) for thrombolysis and 1.35 (95% CI, 1.32–1.48; *p* = 0.047) for thrombectomy. Rates of transfer to stroke unit, compared to pre-pandemic levels, rose significantly, from 57.2% to 62.4% (OR 1.28 [95% CI, 1.23–1.34]; *p* < 0.01), yet without differences across frailty levels. Rates of in-hospital mortality remained unaltered during the pandemic and were not associated with frailty levels, with corresponding pre-pandemic and pandemic levels of 6.3%/6.6% for low frailty patients, 5.8%/6.5% for intermediate, and 12.8%/14.3%

## 4. Discussion

The main finding of this nationwide observational hospital-based study among 101,124 AIS patients in Germany between years 2016 and 2022 is a decreasing trend in frailty that was exacerbated in the early phase of the COVID-19 pandemic. Compared to pre-pandemic levels, frailty-specific alterations in AIS management were observed in rates of thrombolysis and thrombectomy, which increased to a significantly higher degree among low compared to high frailty patients, and in LOS, which decreased among low and intermediate frailty patients but not among those with high frailty levels.

### 4.1. Changes in Frailty Levels

Our findings are in line with previous research demonstrating a decrease in frailty among brain tumor patients during the COVID-19 pandemic in Germany [23]. Interestingly, in patients hospitalized for predominantly degenerative spine pathologies in Germany, the opposite was observed, given that, here, frailty increased during the pandemic [24]. A similar increase in frailty during the pandemic was also described for older patients with heart failure in Japan and older veterans in the US who survived COVID-19 [25,26]. Such disparities in longitudinal frailty trends within the same health system may represent disease-group specific differences in patient flow into hospitals during the pandemic. When interpreting this phenomenon, it is important to note that, during the pandemic, especially in the early phases, in general, total numbers of hospitalizations dropped across all disease groups in most parts of the world, potentially out of fear of hospital acquired COVID-19 both on the part of patients themselves and general practitioners. Other reasons may include pandemic-associated effects on referral patterns, healthcare access, or in-hospital and outpatient triage habits. Such a selection at the thresholds of hospitals may have caused the additional drop-off in frailty levels in in-hospital AIS care observed in our study during the pandemic years, given that there was widespread awareness of an association between frailty and COVID-19 mortality [27]. In addition, pandemic-induced modifications in emergency services may have led to delays between stroke onset and hospital admission during the pandemic that may have prevented frailer AIS patients from reaching hospitals at all or at earlier stages of the disease. Due to the fact that Germany is a country with one of the largest hospital bed capacity per inhabitant in the world and that there were no relevant shut-downs of hospitals during any phase of the pandemic within the nationwide hospital network studied here, it is unlikely that limited access to hospital beds may have relevantly influenced the decreasing total numbers of AIS hospitalizations or even the alteration in frailty levels observed in our cohort.

Of note, in our study, already in pre-pandemic years 2016 through 2019, a longitudinal decrease in total numbers of AIS hospitalizations as well as in frailty was registered. This may be indicative of increased stroke prevention awareness [28] already prior to the pandemic, given the well known associations between risk factors for AIS and frailty [29].

### 4.2. Changes in Baseline Characteristics in Relation to Frailty

The fact that the observed decrease in frailty among AIS patients was not accompanied by changes in patient age supports previous evidence describing age as only one of many contributors to frailty [30]. Nevertheless, in contrast to age, in our study, the burden of comorbidities did show a clear association with frailty. We found that, among high frailty stroke patients, the ECI increased over time, while patients with lower frailty levels showed a decreasing trend. This adds to previous research suggesting a closer link between frailty and comorbidities than with age itself [1].

### 4.3. Changes in Mortality Rates in Relation to Frailty

Given that, in our study, frailty among AIS patients was not associated with in-hospital mortality, our findings are in line with studies reporting no such associations at time of discharge but rather at later points in time [3,7,31,32,33,34,35]. For example, among 433 older patients with AIS at one study center in the UK, frail patients displayed significantly higher 28-day mortality (16.7%), compared to non-frail subjects (5.0%, *p* < 0.01) [32]. Given that the median length of stay in our nationwide cohort ranged between 6 and 7 days, our study’s observational period was about 22 days shorter compared to the UK study, which may account for some of the differences in mortality rates. Furthermore, smaller monocentric studies from large neurovascular centers may be biased in that those centers are likely more specialized in AIS care. They may therefore admit more complex and severe AIS cases, compared to the 78 hospitals including AIS patients throughout Germany in our study. For less severe AIS cases, frailty may play less of a role concerning mortality, independent of whether that is assessed after one or four weeks. Interestingly, rates of transfer to stroke units increased significantly during the study period, yet without link to frailty. This significant increase may have been driven by the decrease in total daily hospital admissions during the pandemic while, at the same time, in general, stroke unit bed capacity remained constant. The fact that stroke unit beds in general were kept in use may have caused the mathematical effect of the observed increase in rates of transfer to stroke units within the smaller total cohort of AIS patients admitted to hospitals during the pandemic.

### 4.4. Changes in LOS in Relation to Frailty

Concerning the relation between frailty and LOS in AIS care our study suggests that, during the pandemic, high frailty AIS patients stayed in hospitals significantly longer compared to pre-pandemic levels, while, at the same time, low and intermediate frailty AIS patients were discharged earlier than before. This may indicate that, among AIS patients examined here, the well-established association between higher frailty and increased LOS was more pronounced during the pandemic era [3,13,36,37]. At the same time, this increase in LOS among high frailty AIS patients may, in parts, also be driven by the observed slight increase in the burden of comorbidity specific to this frailty group. Also, COVID-19 infections among high frailty patients may have caused additional in-hospital stay.

### 4.5. Changes in Rates of Treatment in Relation to Frailty

In our cohort, changes in stroke care during the pandemic were significantly associated with frailty levels in terms of rates of thrombolysis and thrombectomy, which increased to higher degrees among low compared to high frailty AIS patients. Thrombolysis and thrombectomy are applied as early treatments in AIS patients, with thrombectomy being selected in case of large vessel occlusion (LVO), a condition that is associated with higher stroke severity. Given that, within the examined patient cohort, rates of COVID-19 infections were low, such potential infections prior to our study could possibly explain higher rates both of these interventions in low and intermediate frailty patients, as COVID-19 infection was shown to increase stroke risk for up to 28 days [38]. Furthermore, a previously published multinational observational study detected an increase in rates of LVO in AIS with SARS-CoV-2 infection [39]. In addition, one cannot rule out that some degree of selection may have occurred during the pandemic period as AIS therapists may have been less inclined to conduct interventions on high frailty AIS individuals aiming to protect them from potentially increased risk of complication, LOS, and hospital acquired COVID-19. Furthermore, in part, the increase rates in thrombolysis and thrombectomy may have also been influenced by guideline changes around 2018, which increased the time windows for both interventions [40].

### 4.6. The Role of the HFRS in AIS Frailty Assessment

Currently, the majority of large-scale clinical studies on frailty among stroke patients is already using the HFRS as instrument of choice to monitor individual frailty trajectories among AIS patients [3,4,6,7,8]. Our study adds a new aspect to the practical application of the HFRS in AIS care in that it did not examine frailty trajectories but frailty trends based on its cohort-sequential design. Such large scale trend analyses of AIS related frailty align clinical and research aspects of frailty in AIS and are necessary to provide a currently underrepresented perspective in the ongoing discussion of future challenges in AIS care. What makes this even more relevant, is the fact that outcomes of AIS management are only rarely viewed through the lens of frailty, even though, among AIS patients, mortality is expected to increase by 50% and disability-adjusted life-years by 30% by the year 2050 [41,42]. In this context, it is of note that, in our study, over time, frailty trends decreased among hospitalized AIS patients, which suggests that frailty within hospitals may follow trends different from those observed in the out-patient environment, for which increasing trends in frailty over time are well-established. Even though some may argue that the identified median overall decrease in HFRS by about 1 point between prepandemic and pandemic periods may be of limited relevance, it is important to note that the observed decreasing trend in HFRS was consistent over 7 years. This trend is clinically important given its potential to continue over many years and to massively affect in-hospital and out-patient processes in ways that may not be predicted at present. Here, many factors may be at play, for example improved out-patient AIS care for patients with higher levels of frailty.

### 4.7. Limitations

Certain limitations exist in our study. First, routine data evaluation may introduce some misclassification bias (e.g., stroke unit treatment). Second, there was no separate analysis of stroke patients with COVID-19. Third, no individual follow-up data on frailty levels and outcomes were included. Fourth, we acknowledge that the HFRS was originally validated for non-AIS patients aged 75 years and older. Yet, we forewent exclusive analysis on patients of that age cohort given the HFRS’s recent validation in AIS patients of all ages and the associated value for examining changes in in-hospital processes in patients younger than 75 years [43]. Fifth, we did not examine nor adjust for potential regional differences within Germany. Sixth, by the design of the study, we were not able to examine the time frame between AIS onset and presentation to hospitals. Sixth, since we compared two perennial time periods with each other, our study is not able to present more granular continuous frailty trends. Seventh, no decision tree analysis was conducted, which prevents our study from more clearly delineating associations with outcomes and interactions between variables. Finally, given that all data stem exclusively from Germany, the generalizability of our findings to other health systems may be limited. Assessing our results’ generalizability is difficult given that no comparable nationwide studies have been published from other European countries to date. However, regionally limited studies from England, Japan, and Turkey confirm increasing frailty during the pandemic outside of hospitals among older subjects [16,17,44].

## 5. Conclusions

Over the 7-year period examined in this large nationwide hospital-based study in Germany, frailty decreased among AIS patients. These changes in frailty were associated with rates of thrombolysis and thrombectomy and length of stay, but not with patient age or in-hospital mortality.

## Figures and Tables

**Table 1:** Baseline demographics, levels of frailty and comorbidity of stroke patients.

	Prepandemic Period (*n* = 59,377)	Pandemic Period (*n* = 41,747)	Percentage Change Per Frailty Group	*p* †‡
Daily admissions, total, *n* (SD)	40.6 (9.1)	38.4 (8.4)	-	0.01
low frailty (SD)	9.8 (3.9)	10.6 (3.9)	+8.2%	<0.01
intermediate frailty (SD)	20.0 (5.3)	19.2 (5.1)	−4.0%	<0.01
high frailty (SD)	10.8 (3.6)	8.6 (3.2)	−20.4%	ref.
Hospital Frailty Risk Score median (IQR)	9.3 (5.2–15.5)	8.4 (4.4–14.2)	-	<0.01
Age, mean (SD)	73.9 (12.7)	73.9 (12.9)	-	0.25
Sex, male, n (%)	30,783 (51.8)	22,065 (52.9)	-	
female, n (%)	28,594 (48.2)	19,682 (47.1)	-	<0.01
Elixhauser Comorbidity Index, mean (SD)	10.5 (10.3)	9.8 (9.9)	-	<0.01
low frailty	3.1 (6.3)	3.1 (6.0)	0%	0.33
intermediate frailty	9.8 (8.6)	9.6 (8.2)	−2.0%	<0.01
high frailty	18.4 (10.5)	18.6 (10.7)	+1.1%	ref.

SD, standard deviation; IQR, interquartile range. † For the total cohort, statistical comparisons were conducted between pre-pandemic and pandemic values. ‡ Between HFRS groups, changes between pre-pandemic and pandemic values were statistically compared with the high frailty group as reference category.

**Table 2:** Length of in-hospital stay (days) per frailty groups. IQR, interquartile range.

Cohort	Prepandemic Period	Pandemic Peroiod	*p*-Value
Total, median (IQR)	7.0 (5, 13)	6.0 (4, 11)	<0.01
low frailty, median (IQR)	5.0 (4, 7)	4.0 (3, 6)	<0.01
intermediate frailty, median (IQR)	7.0 (5, 11)	6.0 (4, 10)	<0.01
high frailty, median (IQR)	13.0 (7, 20)	13.0 (7, 20)	ref.

**Table 3:** Comparison of treatments and outcomes in different cohorts by frailty groups.

Treatment	Prepandemic Period	Pandemic Period	Odds Ratio (95% CI)	*p* †‡
Thrombolysis, % (*n*)	14.0 (8287)	14.9 (6221)	1.04 (1.00–1.08)	0.053
low frailty, % (*n*)	8.2 (1172)	9.5 (1088)	1.14 (1.02–1.28)	0.020
intermediate frailty, % (*n*)	16.1 (4700)	17,5 (3659)	1.06 (0.98–1.16)	0.158
high frailty, % (*n*)	15.2 (2415)	15.7 (1474)		ref.
Thrombectomy, % (*n*)	5.1 (3021)	6.9 (2871)	1.40 (1.32–1.48)	<0.001
low frailty, % (*n*)	0.5 (75)	1.1 (121)	1.35 (1.00–1.83)	0.047
intermediate frailty, % (*n*)	5.3 (1543)	8.0 (1663)	1.08 (0.96–1.22)	0.204
high frailty, % (*n*)	8.9 (1403)	11.6 (1087)		ref.
Hemicraniectomy, % (*n*)	0.1 (41)	0.1 (42)	1.45 (0.94–2.23)	0.092
low frailty, % (*n*)	0.0 (3)	0.1 (7)	2.38 (0.47–12.05)	0.295
intermediate frailty, % (*n*)	0.1 (28)	0.1 (28)	1.13 (0.38–3.32)	0.828
high frailty, % (*n*)	0.1 (10)	0.1 (7)		ref.
Mechanical ventilation, % (*n*)	4.3 (2575)	4.2 (1751)	0.98 (0.92–1.04)	0.543
low frailty, % (*n*)	0.5 (68)	0.6 (64)	1.06 (0.76–1.50)	0.721
intermediate frailty, % (*n*)	3.2 (950)	3.4 (719)	0.95 (0.84–1.09)	0.482
high frailty, % (*n*)	9.8 (1557)	10.3 (968)		ref.
Stroke unit, % (*n*)	57.2 (32,493)	62.4 (24,954)	1.28 (1.23–1.34)	<0.001
low frailty, % (*n*)	56.2 (7991)	63.3 (7221)	1.06 (0.94–1.19)	0.326
intermediate frailty, % (*n*)	58.3 (16,492)	63.8 (12,864)	1.01 (0.90–1.12)	0.907
high frailty, % (*n*)	56.1 (8010)	57.8 (4869)		ref.
In-hospital mortality, % (*n*)	6.3 (3353)	6.6 (2481)	1.05 (1.00–1.11)	0.070
low frailty, % (*n*)	0.8 (105)	1.0 (115)	1.23 (0.93–1.62)	0.143
intermediate frailty, % (*n*)	5.8 (1542)	6.5 (1227)	1.00 (0.89–1.12)	0.995
high frailty, % (*n*)	12.8 (1706)	14.3 (1139)		ref.

CI, confidence interval. † For the total cohort, statistical comparisons were conducted between pre-pandemic and pandemic values. ‡ Between HFRS groups, changes between pre-pandemic and pandemic values were statistically compared with the high frailty group as reference category. Percentages presented for treatment rates per frailty group represent the proportion of each type of treatment within each frailty group separately, therefore not adding up to 100%.

## Data Availability

The original contributions presented in this study are included in the article/Supplementary Material. Further inquiries can be directed to the corresponding author.

## Supplementary Materials

The following supporting information can be downloaded at: https://www.mdpi.com/article/10.3390/neurosci6030088/s1, Table S1: Frailty levels per age group and prevalence of female sex per frailty levels.

## References

[1] Fried L.P., Tangen C.M., Walston J., Newman A.B., Hirsch C., Gottdiener J., Seeman T., Tracy R., Kop W.J., Burke G. (2001). Frailty in older adults: Evidence for a phenotype. J. Gerontol. A. Biol. Sci. Med. Sci..

[2] Taylor-Rowan M., Hafdi M., Drozdowska B., Elliott E., Wardlaw J., Quinn T.J. (2023). Physical and brain frailty in ischaemic stroke or TIA: Shared occurrence and outcomes. A cohort study. Eur. Stroke. J..

[3] Kilkenny M.F., Phan H.T., Lindley R.I., Kim J., Lopez D., Dalli L.L., Grimley R., Sundararajan V., Thrift A.G., Andrew N.E. (2021). Utility of the Hospital Frailty Risk Score Derived from Administrative Data and the Association with Stroke Outcomes. Stroke.

[4] Burton J.K., Stewart J., Blair M., Oxley S., Wass A., Taylor-Rowan M., Quinn T.J. (2022). Prevalence and implications of frailty in acute stroke: Systematic review & meta-analysis. Age Ageing.

[5] Palmer K., Vetrano D.L., Padua L., Romano V., Rivoiro C., Scelfo B., Marengoni A., Bernabei R., Onder G. (2019). Frailty Syndromes in Persons With Cerebrovascular Disease: A Systematic Review and Meta-Analysis. Front. Neurol..

[6] Pinho J., Küppers C., Nikoubashman O., Wiesmann M., Schulz J.B., Reich A., Werner C.J. (2021). Frailty is an outcome predictor in patients with acute ischemic stroke receiving endovascular treatment. Age Ageing.

[7] Schnieder M., Bähr M., Kirsch M., Maier I., Behme D., Riedel C.H., Psychogios M.N., Brehm A., Liman J., von Arnim C.A.F. (2021). Analysis of Frailty in Geriatric Patients as a Prognostic Factor in Endovascular Treated Patients with Large Vessel Occlusion Strokes. J. Clin. Med..

[8] Zhang W., Anderson C.S., Kilkenny M.F., Kim J., Dewey H.M., Andrew N.E., Lannin N.A., Thrift A.G., Grimley R., Sundararajan V. (2020). Hospital admissions prior to primary intracerebral haemorrhage and relevant factors associated with survival. J. Stroke Cerebrovasc. Dis..

[9] Gilbert T., Neuburger J., Kraindler J., Keeble E., Smith P., Ariti C., Arora S., Street A., Parker S., Roberts H.C. (2018). Development and validation of a Hospital Frailty Risk Score focusing on older people in acute care settings using electronic hospital records: An observational study. Lancet.

[10] Van Nguyen T., Tran H.M., Ngo T.K.T. (2025). Comparative clinical frailty scale and hospital frailty risk score in identifying frailty and predicting mid-term outcomes in older patients with acute coronary syndrome: A multicenter cohort study in Vietnam. BMC Geriatr..

[11] Yamamoto Y., Hori S., Ushida K., Shirai Y., Shimizu M., Kato Y., Momosaki R. (2024). Impact of Frailty Risk on Functional Outcome after Aneurysmal Subarachnoid Hemorrhage: A Historical Cohort Study. Neurol. Med. Chir. Tokyo..

[12] Renedo D., Acosta J.N., Koo A.B., Rivier C., Sujijantarat N., de Havenon A., Sharma R., Gill T.M., Sheth K.N., Falcone G.J. (2023). Higher Hospital Frailty Risk Score is Associated with Increased Risk of Stroke: Observational and Genetic Analyses. Stroke.

[13] Kim D., Yang P.S., Sung J.H., Jang E., Yu H.T., Kim T.H., Uhm J.S., Kim J.Y., Pak H.N., Lee M.H. (2022). Effectiveness and Safety of Anticoagulation Therapy in Frail Patients with Atrial Fibrillation. Stroke.

[14] United Nations, Department of Economic and Social Affairs, Population Division 2022 World Population Prospects 2022; World Population Prospects 2022: Summary of Results. https://www.un.org/development/desa/pd/sites/www.un.org.development.desa.pd/files/wpp2022_summary_of_results.pdf.

[15] Dengler J., Prass K., Palm F., Hohenstein S., Pellisier V., Stoffel M., Hong B., Meier-Hellmann A., Kuhlen R., Bollmann A. (2022). Changes in nationwide in-hospital stroke care during the first four waves of COVID-19 in Germany. Eur. Stroke J..

[16] Garner I.W., Varey S., Navarro-Pardo E., Marr C., Holland C.A. (2022). An observational cohort study of longitudinal impacts on frailty and well-being of COVID-19 lockdowns in older adults in England and Spain. Health Soc. Care Community.

[17] Yamada M., Kimura Y., Ishiyama D., Otobe Y., Suzuki M., Koyama S., Kikuchi T., Kusumi H., Arai H. (2021). The Influence of the COVID-19 Pandemic on Physical Activity and New Incidence of Frailty among Initially Non-Frail Older Adults in Japan: A Follow-Up Online Survey. J. Nutr. Health Aging..

[18] Nachtigall I., Lenga P., Jóźwiak K., Thürmann P., Meier-Hellmann A., Kuhlen R., Brederlau J., Bauer T., Tebbenjohanns J., Schwegmann K. (2020). Clinical course and factors associated with outcomes among 1904 patients hospitalized with COVID-19 in Germany: An observational study. Clin. Microbiol. Infect..

[19] Moore B., White S., Wahington R., Coenen N., Elixhauser A. (2017). Identifying increased risk of readmission and in-hospital mortality using hospital administrative data. Med. Care.

[20] Baayen R.H., Davidson D.J., Bates D.M. (2008). Mixed-Effects modeling with crossed random effects for subjects and items. J. Mem. Lang..

[21] Bates D., Mächler M., Bolker B., Walker S. (2015). Fitting linear mixed-effects models using Lme4. J. Stat. Softw..

[22] R Core Team (2019). R: A Language and Environment for Statistical Computing.

[23] Hong B., Allam A., Heese O., Gerlach R., Gheewala H., Rosahl S.K., Stoffel M., Ryang Y.M., Burger R., Carl B. (2023). Trends in frailty in brain tumor care during the COVID-19 pandemic in a nationwide hospital network in Germany. Eur. Geriatr. Med..

[24] Dengler J., Gheewala H., Kraft C.N., Hegewald A.A., Dörre R., Heese O., Gerlach R., Rosahl S., Maier B., Burger R. (2024). Changes in frailty among patients hospitalized for spine pathologies during the COVID-19 pandemic in Germany-a nationwide observational study. Eur. Spine J..

[25] Kato M., Ono S., Seko H., Tsukamoto T., Kurita Y., Kubo A., Omote T., Omote S. (2021). Trajectories of frailty, physical function, and physical activity levels in elderly patients with heart failure: Impacts of interruption and resumption of outpatient cardiac rehabilitation due to COVID-19. Int. J. Rehabil. Res..

[26] Seligman B., Wysham K.D., Shahoumian T., Orkaby A.R., Goetz M.B., Osborne T.F., Smith V.A., Maciejewski M.L., Hynes D.M., Boyko E.J. (2024). Change in frailty among older COVID-19 survivors. J. Am. Geriatr. Soc..

[27] Hewitt J., Carter B., Vilches-Moraga A., Quinn T.J., Braude P., Verduri A., Pearce L., Stechman M., Short R., Price A. (2020). The effect of frailty on survival in patients with COVID-19 (COPE): A multicentre, European, observational cohort study. Lancet Public Health.

[28] Yafasova A., Fosbøl E.L., Christiansen M.N., Vinding N.E., Andersson C., Kruuse C., Johnsen S.P., Gislason G.H., Torp-Pedersen C., Køber L. (2020). Time trends in incidence, comorbidity, and mortality of ischemic stroke in Denmark (1996–2016). Neurology.

[29] Veronese N. (2020). Frailty as Cardiovascular Risk Factor (and Vice Versa). Adv. Exp. Med. Biol..

[30] Hoogendijk E.O., Afilalo J., Ensrud K.E., Kowal P., Onder G., Fried L.P. (2019). Frailty: Implications for clinical practice and public health. Lancet.

[31] Kim M.G., Gandhi C., Azizkhanian I., Epstein B., Mittal A., Lee N., Santarelli J., Schmidt M., Al-Mufti F., Bowers C.A. (2020). Frailty and spontaneous intracerebral hemorrhage: Does the modified frailty index predict mortality?. Clin. Neurol. Neurosurg..

[32] Evans N.R., Wall J., To B., Wallis S.J., Romero-Ortuno R., Warburton E.A. (2020). Clinical frailty independently predicts early mortality after ischaemic stroke. Age Ageing.

[33] Imaoka Y., Kawano T., Hashiguchi A., Fujimoto K., Yamamoto K., Nishi T., Otsuka T., Yano S., Mukasa A. (2018). Modified frailty index predicts postoperative outcomes of spontaneous intracerebral hemorrhage. Clin. Neurol. Neurosurg..

[34] Joyce N., Atkinson T., Mc Guire K., Wiggam M.I., Gordon P.L., Kerr E.L., Patterson C.E., McILmoyle J., Roberts G.E., Flynn P.A. (2022). Frailty and stroke thrombectomy outcomes-an observational cohort study. Age Ageing.

[35] Taylor-Rowan M., Keir R., Cuthbertson G., Shaw R., Drozdowska B., Elliott E., Evans J., Stott D., Quinn T.J. (2019). Pre-Stroke Frailty is Independently Associated with Post-Stroke Cognition: A Cross-Sectional Study. J. Int. Neuropsychol. Soc..

[36] Noguchi M., Kubo H., Kanai M., Nozoe M., Shimada S. (2021). Relationship between pre-stroke frailty status and short-term functional outcome in older patients with acute stroke-A mediation analysis. Arch. Gerontol. Geriatr..

[37] Seamon B.A., Simpson K.N. (2019). The Effect of Frailty on Discharge Location for Medicare Beneficiaries After Acute Stroke. Arch. Phys. Med. Rehabil..

[38] Katsoularis I., Fonseca-Rodríguez O., Farrington P., Lindmark K., Fors Connolly A.M. (2021). Risk of acute myocardial infarction and ischaemic stroke following COVID-19 in Sweden: A self-controlled case series and matched cohort study. Lancet.

[39] Shahjouei S., Anyaehie M., Koza E., Tsivgoulis G., Naderi S., Mowla A., Avula V., Vafaei Sadr A., Chaudhary D., Farahmand G. (2021). SARS-CoV-2 Is a Culprit for Some, but Not All Acute Ischemic Strokes: A Report from the Multinational COVID-19 Stroke Study Group. J. Clin. Med..

[40] Powers W.J., Rabinstein A.A., Ackerson T., Adeoye O.M., Bambakidis N.C., Becker K., Biller J., Brown M., Demaerschalk B.M., Hoh B. (2018). American Heart Association Stroke Council. 2018 Guidelines for the Early Management of Patients with Acute Ischemic Stroke: A Guideline for Healthcare Professionals from the American Heart Association/American Stroke Association. Stroke.

[41] Feigin V.L., Owolabi M.O., World Stroke Organization–Lancet Neurology Commission Stroke Collaboration Group (2023). Pragmatic solutions to reduce the global burden of stroke: A World Stroke Organization-Lancet Neurology Commission. Lancet Neurol..

[42] Schlögl M., Quinn T.J., International Stroke Recovery and Rehabilitation Alliance (SRRRA) Frailty Stroke Group (2024). Pragmatic solutions for the global burden of stroke. Lancet Neurol..

[43] Kutrani H., Briggs J., Prytherch D., Spice C. (2025). Using the Hospital Frailty Risk Score to predict length of stay across all adult ages. PLoS ONE.

[44] Mete B., Tanır F., Demirhindi H., İnaltekin A., Kanat C. (2022). Impact of the COVID-19 Pandemic on Frailty in Older Adults. Eur. J. Geriatric. Gerontol..

