# Supplementary material for: Frailty in Stroke Care in Germany Between 2016 and 2022—A Retrospective, Hospital-Based Nationwide Cohort Study
Source: NeuroSci. 2025 Sep 8;6(3):88. doi: 10.3390/neurosci6030088 (PMC12452760; doi:10.3390/neurosci6030088)
Supplement: Supplementary file 1 [file neurosci-06-00088-s001.zip › neurosci-3761818-supplementary.pdf]

Table S1. Frailty levels per age group and prevalence of female sex per frailty levels.

	Prepandemic period	Pandemic period	p-value
**Age groups (from 55 years upward)**			
**55-64 years**			
low frailty, n (%)	3,007 (36.1)	2,386 (39.3)	<0.01
intermediate frailty, n (%)	4,345 (52.2)	3,095 (51.0)	0.16
high frailty, n (%)	970 (11.7)	585 (9.6)	<0.01
**65-74 years**			
low frailty, n (%)	3,604 (28.9)	2,996 (32.5)	<0.01
intermediate frailty, n (%)	6,547 (52.5)	4,673 (50.7)	0.01
high frailty, n (%)	2,330 (18.7)	1,543 (16.7)	<0.01
**75-84 years**			
low frailty, n (%)	4,398 (20.0)	3,416 (23.6)	<0.01
intermediate frailty, n (%)	10,722 (48.8)	7,333 (50.6)	<0.01
high frailty, n (%)	6,830 (31.1)	3,735 (25.8)	<0.01
**>85 years**			
low frailty, n (%)	1,072 (9.1)	1,115 (12.8)	<0.01
intermediate frailty, n (%)	5,336 (45.5)	4,264 (49.1)	<0.01
high frailty, n (%)	5,319 (45.4)	3,309 (38.1)	<0.01
**Female sex**			
low frailty, n (%)	5,851 (41.0)	4,666 (40.7)	0.40
intermediate frailty, n (%)	13,823 (47.2)	9,831 (47.1)	0.25
high frailty, n (%)	8,920 (56.3)	5,185 (55.2)	ref.
